# A comparative analysis of predictive models of morbidity in intensive care unit after cardiac surgery – Part II: an illustrative example

**DOI:** 10.1186/1472-6947-7-36

**Published:** 2007-11-22

**Authors:** Gabriele Cevenini, Emanuela Barbini, Sabino Scolletta, Bonizella Biagioli, Pierpaolo Giomarelli, Paolo Barbini

**Affiliations:** 1 Department of Surgery and Bioengineering, University of Siena, Siena, Italy; 2 Department of Physiopathology, Experimental Medicine and Public Health, University of Siena, Siena, Italy

## Abstract

**Background:**

Popular predictive models for estimating morbidity probability after heart surgery are compared critically in a unitary framework. The study is divided into two parts. In the first part modelling techniques and intrinsic strengths and weaknesses of different approaches were discussed from a theoretical point of view. In this second part the performances of the same models are evaluated in an illustrative example.

**Methods:**

Eight models were developed: Bayes linear and quadratic models, *k*-nearest neighbour model, logistic regression model, Higgins and direct scoring systems and two feed-forward artificial neural networks with one and two layers. Cardiovascular, respiratory, neurological, renal, infectious and hemorrhagic complications were defined as morbidity. Training and testing sets each of 545 cases were used. The optimal set of predictors was chosen among a collection of 78 preoperative, intraoperative and postoperative variables by a stepwise procedure. Discrimination and calibration were evaluated by the area under the receiver operating characteristic curve and Hosmer-Lemeshow goodness-of-fit test, respectively.

**Results:**

Scoring systems and the logistic regression model required the largest set of predictors, while Bayesian and *k*-nearest neighbour models were much more parsimonious. In testing data, all models showed acceptable discrimination capacities, however the Bayes quadratic model, using only three predictors, provided the best performance. All models showed satisfactory generalization ability: again the Bayes quadratic model exhibited the best generalization, while artificial neural networks and scoring systems gave the worst results. Finally, poor calibration was obtained when using scoring systems, *k*-nearest neighbour model and artificial neural networks, while Bayes (after recalibration) and logistic regression models gave adequate results.

**Conclusion:**

Although all the predictive models showed acceptable discrimination performance in the example considered, the Bayes and logistic regression models seemed better than the others, because they also had good generalization and calibration. The Bayes quadratic model seemed to be a convincing alternative to the much more usual Bayes linear and logistic regression models. It showed its capacity to identify a minimum core of predictors generally recognized as essential to pragmatically evaluate the risk of developing morbidity after heart surgery.

## Background

The increasing number of diagnostic and therapeutic choices and the demand for quality and cost control have contributed to a proliferation of techniques of pattern recognition and decision making in all biomedical fields. In recent years, many different models have been proposed for the prediction of adverse outcome in heart surgery patients [1-6]. This prompted us to critically analyse the features of a number of popular systems for predicting patient morbidity in the cardiac postoperative intensive care unit (ICU), in a unitary framework.

The study is divided into two parts. In the first part different methods for estimating morbidity probability were grouped into categories according to the underlying mathematical principles. Eight predictive models, based on the Bayes rule [6-9], *k*-nearest neighbour [7,10], logistic regression [11], integer score systems [3,6] and artificial neural networks [12,13], were investigated from a theoretical point of view. Modelling techniques and intrinsic strengths and weaknesses of each predictive model were analysed and discussed in view of clinical applications.

Although knowledge of theoretical features, strengths and weaknesses of different approaches are fundamental for developing a predictive model of morbidity in the ICU, the final choice of model also has to consider the context and clinical scenario where the model will be used. Actual performances of locally-developed competitive models have to be evaluated and compared using real experimental data in order to reconcile local needs and model response. In this second part of the study, the experimental performance of the previously analysed models in predicting the risk of morbidity is evaluated in a real clinical scenario. All models were developed and tested using preoperative, intraoperative and postoperative data acquired in heart surgery patients. Since the aim of this study was to experimentally test the performance of a number of popular predictive models when locally customized to a specific scenario, not to develop a generally applicable model (for example, for benchmarking purposes), both training and testing data was acquired in the same postoperative cardiac ICU and the models were not tested on other independent data collected in different ICUs. Discrimination, generalization, calibration, simplicity of use and updating were the criteria used to assess differences between them, taking the specialised ICU as an illustrative example.

## Methods

### Sample set and variable collection

We considered data acquired in the whole set of 1090 patients who underwent coronary artery bypass grafting and were admitted to the intensive care unit of the Department of Surgery and Bioengineering of Siena University between 1^st ^January 2002 and 31^st ^December 2004. Standard preoperative and postoperative management and cardiopulmonary bypass (CPB) were performed [14]. The study was approved by the Ethics Committee of our institution.

A collection of 78 preoperative, intraoperative and postoperative variables were considered as likely risk predictors, that could be associated with morbidity development in the ICU. A dichotomous (binary) variable was chosen as ICU outcome (morbidity). Preoperative and intraoperative data was collected under the anaesthesiologist's supervision. Postoperative data was collected in the first three hours after admission to the ICU, except the binary outcome that was retrieved from medical records after discharge from the ICU. In total, 48 preoperative, intraoperative and postoperative continuous variables (Tables 1, 2 and 3, respectively) and 31 dichotomous variables (Tables 4, 5, 6) were used.

**Table 1:** Preoperative continuous variables

**Acronym**	**Description (units)**	**Training set**		**Testing set**		**Cut-off**
		**Mean**	**SD**	**Mean**	**SD**	
Age	Age (years)	67.5	8.9	67.5	8.9	71↑
H	Height (cm)	167	8.0	167	7.5	n.s.
W	Weight(kg)	72.4	11	72.8	12	72↓
BSA	Body surface area (m^2^)	1.79	0.17	1.80	0.17	1.8↓
Pre-HCT	Hematocrit (%)	29.7	4.6	29.9	4.2	n.s.
Cr	Creatinine (mg/l)	1.09	0.51	1.07	0.38	1.2↑
Alb	Albumin (g/l)	3.86	0.44	3.85	0.41	n.s.
Bil	Bilirubin (mg/dl)	0.81	0.40	0.83	0.30	n.s.
Pre-CI	Cardiac index (l/min/m^2^)	2.71	0.69	2.70	0.69	n.s.
Pre-PaCO_2_	Partial pressure of arterial CO_2 _(mmHg)	34.0	4.5	34.2	4.6	n.s.

↑ or ↓: increased morbidity risk for values greater or less than cut-off, respectively (n.s.: not statistically significant); SD, standard deviation.

**Table 2:** Intraoperative continuous variables

**Acronym**	**Description (units)**	**Training set**		**Testing set**		**Cut-off**
		**Mean**	**SD**	**Mean**	**SD**	
Xclampt	Aortic clamp time (min)	79.3	32	79.2	34	90↑
CPBt	Cardio-pulmonary bypass time (min)	113	42	115	49	120↑
HR-end	Heart rate at end of surgery (min^-1^)	91.3	13	91.7	12	100↑
Intra-CI	Cardiac index (l/min/m^2^)	2.67	0.74	2.68	0.70	n.s.
Diur	Diuresis (cl/hour)	166	82	169	84	n.s.
TBU	Transfused blood (ml)	67.8	216	67.2	216	300↑
HR-ICU	Heart rate at ICU arrival (min^-1^)	91.3	13	91.7	12	100↑

↑ or ↓: increased morbidity risk for values greater or less than cut-off, respectively (n.s.: not statistically significant); SD, standard deviation; ICU, intensive care unit.

**Table 3:** Postoperative continuous variables

**Acronym**	**Description (units)**	**Training set**		**Testing set**		**Cut-off**
		**Mean**	**SD**	**Mean**	**SD**	
SAP	Systolic arterial pressure (mmHg)	132	23	130	24	n.s.
DAP	Diastolic arterial pressure (mmHg)	70.2	14	69.8	13	n.s.
MAP	Mean arterial pressure (mmHg)	90.9	16	90.2	16	n.s.
CVP	Central venous pressure (mmHg)	8.59	3.4	8.57	3.3	n.s.
FiO_2_	Fraction of inspired O_2_	0.533	0.072	0.530	0.075	n.s.
pH	Potential of hydrogen	7.45	0.06	7.45	0.06	n.s.
PaCO_2_	Partial pressure of arterial CO_2 _(mmHg)	35.3	5.4	35.5	5.3	n.s.
HCO_3_	HCO_3 _arterial level (mmol/l)	24.9	2.3	25.0	2.3	n.s.
AaO_2_	Alveolar-arterial O_2 _gradient (mmHg)	187	65	188	69	n.s.
Post-HCT	Hematocrit (%)	29.7	4.6	29.9	4.2	n.s.
K	Potassium (mEq/l)	4.05	0.49	4.06	0.50	5↑
Gly	Glycaemia (mmol/l)	170	54	172	54	n.s.
Temp	Body temperature (°C)	35.4	0.89	35.3	0.92	n.s.
WBC	White blood cells (nl^-1^)	12.2	4.5	12.6	4.3	n.s.
Hb	Hemoglobin (g/dl)	9.85	1.5	9.90	1.4	n.s.
PaO_2_	Partial pressure of arterial O_2 _(mmHg)	149	39	145	40	n.s.
SaO_2_	Arterial oxygen saturation (%)	98.5	1.6	98.4	1.7	n.s.
PvO_2_	Partial pressure of venous O_2 _(mmHg)	32.7	4.6	33.1	4.7	32↓
SvO_2_	Venous O_2 _saturation (%)	63.5	6.9	64.1	7.0	62.5↓
CaO_2_	Arterial O_2 _content (ml/dl)	13.0	1.9	13.0	1.8	n.s.
CvO_2_	Venous O_2 _content (ml/dl)	8.41	1.7	8.54	1.7	8↓
AVO_2_	Artero-venous O_2 _difference (ml/dl)	4.60	1.0	4.52	1.0	5↑
Post-CI	Cardiac index at ICU (l/min/m^2^)	2.63	0.65	2.61	0.66	2.5↓
SVI	Stroke volume index (ml/m^2^)	29.4	8.2	29.0	8.2	28↓
DO_2_I	O_2 _delivery index (ml/min/m^2^)	344	93	344	97	320↓
O_2_ER	O_2 _extraction ratio (%)	35.3	7.0	34.6	7.2	40↑
RI	Respiratory Index (AaO_2_/PaO_2_)	1.46	0.93	1.52	1.01	n.s.
P/F	PaO_2_/FiO_2 _ratio (mmHg)	283	81	280	85	n.s.
VCO_2_	CO_2 _production (ml/min)	191	50	187	50	200↓
VO_2_	O_2 _consumption (ml/min)	224	59	219	59	220↓
SVRI	Systemic vascular resistance index (MPa·s/m)	265	83	265	85	280↑

↑ or ↓:increased morbidity risk for values greater or less than cut-off, respectively (n.s.: not statistically significant); SD, standard deviation; ICU, intensive care unit.

**Table 4:** Preoperative dichotomous variables

**Acronym**	**Description**	**Training set**		**Testing set**	
		**N**	**%**	**N**	**%**
Gender	Sex (female)	141	25.9	133	24.4
COPD	Chronic obstructive pulmonary disease	51	9.4	46	8.4
PAH	Pulmonary artery hypertension	10	1.8	17	3.1
Arrhy	Arrhythmia	77	14.1	81	14.9
CHF	Congestive heart failure	24	4.4	31	5.7
PVD	Peripheral vascular disease	122	22.4	98	18.0
TIA	Transient ischemic attacks	34	6.2	21	3.9
PVS	Previous vascular surgery	49	9.0	42	7.7
LMSS	Left main stem stenosis	343	62.9	332	60.9
Endoc	Endocarditis	1	0.2	2	0.4
Pre-IABP	Intra aortic balloon pump	14	2.6	13	2.4
AMI-1m	Acute myocardial infarction within a month	124	22.8	114	20.9
Diab	Diabetes	104	19.1	94	17.2
REDO-1	One previous heart operation	10	1.8	19	3.5
REDO-2	Two previous heart operations	2	0.4	0	0.0
LVEF-35%	Left ventricular ejection fraction < 35%	52	9.5	47	8.6
EM	Emergency	42	7.7	51	9.4

**Table 5:** Intraoperative dichotomous variables

**Acronym**	**Description**	**Training set**		**Testing set**	
		**N**	**%**	**N**	**%**
MVR	Mitral valve replaced with artificial valve	12	2.2	8	1.5
MR	Mitral valve repaired surgically	10	1.8	10	1.8
AVR	Aortic valve replaced with artificial valve	32	5.9	39	7.2
TVR	Tricuspid valve repaired surgically	1	0.2	0	0.0
CABG-A	Coronary artery bypass graft and aortic surgery	18	3.3	15	2.8
CABG-C	Coronary artery bypass graft and carotid surgery	13	2.4	13	2.4
LIMA	Coronary bypass with left internal mammary artery	440	80.7	431	79.1
IABP	Intra-aortic balloon pump	12	2.2	9	1.7
Xclamp	Two or more clampings of ascending aorta	7	1.3	10	1.8

**Table 6:** Postoperative dichotomous variables

**Acronym**	**Description**	**Training set**		**Testing set**	
		**N**	**%**	**N**	**%**
Card-ID	Cardiac inotropic drugs	74	13.6	69	12.7
VD	Vasodilator drugs	298	54.7	323	59.3
AD	Antiarrhythmic drugs	27	5.0	29	5.3
IABP-ICU	Intra-aortic balloon pump in intensive care unit	14	2.6	10	1.8
M	Morbidity (outcome)	113	20.7	113	20.7

Cardiopulmonary bypass time (Table 2) was the total of all bypass runs if a second or subsequent period of cardiopulmonary bypass was conducted. Re-operations (Table 4) were considered as separate variables in the analysis [3].

According to the definitions of Higgins and colleagues [3,15], emergency cases (Table 4) were defined as unstable angina, unstable hemodynamics, or ischemic valve dysfunction that could not be controlled medically. Left ventricular ejection fractions less than 35% were considered severely impaired (Table 4). Diabetes or chronic obstructive pulmonary disease (Table 4) were diagnosed only if the patient was maintained on appropriate medication.

Data was ranked chronologically on the basis of patient hospital discharge and organized in a database. The database was divided into two sets of equal size (545 cases each): a training set consisting of patients in odd positions in the original ranked database and a testing set consisting of the other patients, that is, those in even positions in the original database. To ensure that alternate allocation of cases did not introduce systematic sampling errors, training and testing data was compared using the Fisher exact test for dichotomous variables and the z-test or Mann-Whitney test for continuous normally or non-normally distributed variables, respectively [16]. Normality was assessed by the Kolmogorov-Smirnov test [16]. No significant difference was found between training and testing data, setting statistical significance at a p-value less than 0.05. Tables 1, 2, 3, 4, 5, 6 summarize the descriptive statistics of the training and test sets: continuous variables were described by means and standard deviations and dichotomous variables by frequencies and percentages.

Tables 1, 2, 3 also show the cut-off values at which continuous variables were dichotomised to develop integer score systems. They were chosen by setting sensitivity (SE) and specificity (SP) equal and testing the confidence interval for the odds ratio (for details see Section "Model description" in PartI of the present study). In the table, n.s. means that the odds ratio of the dichotomised variable was not significantly greater than 1.

Morbidity outcome was defined for patients developing at least one of the following clinical complications.

*Cardiovascular complications*: myocardial infarction (documented by electrocardiography and enzyme criteria); low cardiac output (requiring inotropic support for more than 24 hours, intraaortic balloon pump or ventricular assist device); or severe arrhythmias (requiring treatment or cardiopulmonary resuscitation).

*Respiratory complications*: prolonged ventilatory support (mechanical ventilatory support for more than 24 hours); re-intubation; tracheostomy; or clinical evidence of pulmonary embolism, edema or adult respiratory distress syndrome.

*Neurological (central nervous system) complications*: focal brain lesion confirmed by clinical findings and/or computed tomography; diffuse encephalopathy with more than 24 hours of severely altered mental status; or unexplained failure to awaken within 24 hours after operation.

*Renal complications*: acute renal failure needing dialysis

*Infectious complications*: culture-proven pneumonia; mediastinitis; wound infection; septicaemia with appropriate clinical findings; or septic shock.

*Hemorrhagic complications*: bleeding requiring re-operation

Note that the above outcome definition implies a compound endpoint of morbidity. This extensive definition is widely used when models for predicting major adverse outcomes are employed in ICU [3], although it limits the power of any single model to predict who gets a specific complication. On the other hand, it allows the number of events to be increased (the morbidity percentage in the whole patient set considered here was 20.7%) and the contribution of patient management to outcome is more evident when the endpoint occurs more frequently.

### Predictive model development

The following models were developed locally to predict morbidity probability: Bayesian linear (BL) model, Bayesian quadratic (BQ) model, *k*-nearest neighbour (*k*NN) model, logistic regression (LR) model, Higgins score (HS) model derived from the previous LR model, direct score (DS) model, and two feed-forward artificial neural networks (ANNs) with one and two layers (ANN1 and ANN2, respectively). The theoretical details of the models were described in PartI of the study.

The above training and testing sets of 545 cases were used to train and test all models. Briefly, model development included: feature selection; evaluation of discrimination performance by AUC, that is area under the receiver operating curve (ROC); assessment of calibration by Hosmer-Lemeshov (HL) goodness-of-fit test using Ĉ-statistics; evaluation of accuracy by mean squared error (MSE); recalibration of model-predicted probabilities when necessary.

Artificial neural networks were trained using a batch training method which updates neural weights and biases after all training patterns have been processed, that is, after each epoch. An iterative training algorithm with gradient descendent momentum and adaptive learning rate was used to minimize MSE. The influence of initialization on the solution was reduced by always performing 99 training sessions starting from 99 different randomly-selected initial conditions; the 99 corresponding values obtained for AUC were sorted from lowest to highest and the results of the session giving the 50^th ^value of AUC were taken.

After the stepwise feature selection was performed for each predictive model on the training data by means of proper techniques such as leave-one-out, the 95% confidence interval of AUC and its median value (AŨC) were estimated for every set of selected features using 1000 different random samples generated by the bootstrap resampling method in the training and testing sets. The same samples were used to compare AUC values of different models in test data, by performing a Wilcoxon matched-pairs signed-ranks test [16].

When applying stepwise feature selection on training data to a model, techniques, such as leave-one-out, may not ensure satisfactory generalization. The final selection of the number of features used for predicting morbidity was therefore made trading discrimination capacity off against model complexity on the bootstrap samples of testing data (that is, on data not employed in the training process). The behaviour of AUC was first analysed in these test samples in relation to the set of features selected step-by-step by the previous stepwise procedure and the number of feature (*d*_*M*_) allowing the maximum value of AŨC was taken as reference point. Then AUC values obtained with a number of selected features less than *d*_*M *_were compared to those of *d*_*M *_by the Wilcoxon matched-pairs signed-ranks test. Finally, the optimal number of selected features was chosen as the minimum number ensuring no significant difference in AUC (0.05 probability) with respect to *d*_*M*_. Of course, if all comparisons gave significant differences, *d*_*M *_was chosen as the optimal number of selected features to be used for predicting morbidity.

Once optimized to ensure suitable generalization with the best discrimination performance, models with inadequate calibration were recalibrated by applying a cubic monotonic transformation (see Part I of the study) to the ranked predicted probabilities, so as to reach a more reliable estimation of morbidity probability.

All computer calculations were performed by means of locally-developed specific codes written in the Matlab programming language using the statistics and optimization toolboxes [17].

## Results

For all models, Table 7 shows the predictor variables entered step-by-step or removed during the stepwise feature selection process. Variables that were removed appear in square brackets.

**Table 7:** Variables entered and removed (in square brackets) at each step of the stepwise selection procedure

**Step no**.	**BL**	**BQ**	***k*NN**	**LR**	**HS**	**DS**	**ANN1**	**ANN2**
1	O_2_ER	O_2_ER	Post-CI	O_2_ER	O_2_ER	SvO_2_	O_2_ER	O_2_ER
2	VCO_2_	DO_2_I	O_2_ER	VCO_2_	VCO_2_	Card-ID	Card-ID	VO_2_
3	Card-ID	Card-ID	Card-ID	Card-ID	Card-ID	DO_2_I	VO_2_	Card-ID
4	PVD	PVD	PVD	PVD	PVD	O_2_ER	PVD	PVD
5	TBU	W	TBU	TBU	TBU	PVD	TBU	Gly
6	EM	VD	MAP	EM	EM	O_2_ER	Pre-CI	Gender
7	Pre-CI	DAP		SAP	SAP	EM	EM	MVR
8	WBC	SAP		Pre-CI	Pre-CI	BSA	WBC	Cr
9	SAP	Diur		WBC	WBC	AD	Age	AVO_2_
10	Age	Xclamp		SaO_2_	SaO_2_	CHF	SaO_2_	Arrhy
11	PvO_2_	H		PvO_2_	PvO_2_	MVR	AD	
12	SaO_2_	Gender		AD	AD	O_2_ER	P/F	
13	AD			PaO_2_	PaO_2_	MR	PVS	
14	SvO_2_			Cr	Cr	EM	Temp	
15	Xclamp			CvO_2_	CvO_2_	Card-ID		
16	PaO_2_			Xclamp	Xclamp	O_2_ER		
17	[PvO_2_]			DO_2_I	DO_2_I	PVD		
18	Cr			W	W	Diab		
19	Intra-CI			SVRI	SVRI	VCO_2_		
20	Post-CI			[VCO_2_]	[VCO_2_]	O_2_ER		
21	[Age]			Pre-IABP	Pre-IABP	AD		
22	W			CHF	CHF	O_2_ER		
23	[Pre-CI]			[Xclamp]	[Xclamp]	CHF		
24	DO_2_I					CABG-C		
25	[VCO_2_]					MR		
26	Bil					PAH		
27	Hb					O_2_ER		
28	[WBC]					IABP		
29	SVRI					O_2_ER		
30	[Post-CI]					IABP		
31	BSA					O_2_ER		
32	CABG-A					PVS		
33						IABP		
34						O_2_ER		
35						IABP		

BL, Bayes linear model; BQ, Bayes quadratic model; *k*NN, *k*-nearest neighbour model; LR, logistic regression model; HS, Higgins score system; DS, direct score system; ANN1, one-layer artificial neural network; ANN2, two-layer artificial neural network. Predictor variable abbreviations are indicated in Tables 1-6.

**Figure 1:**
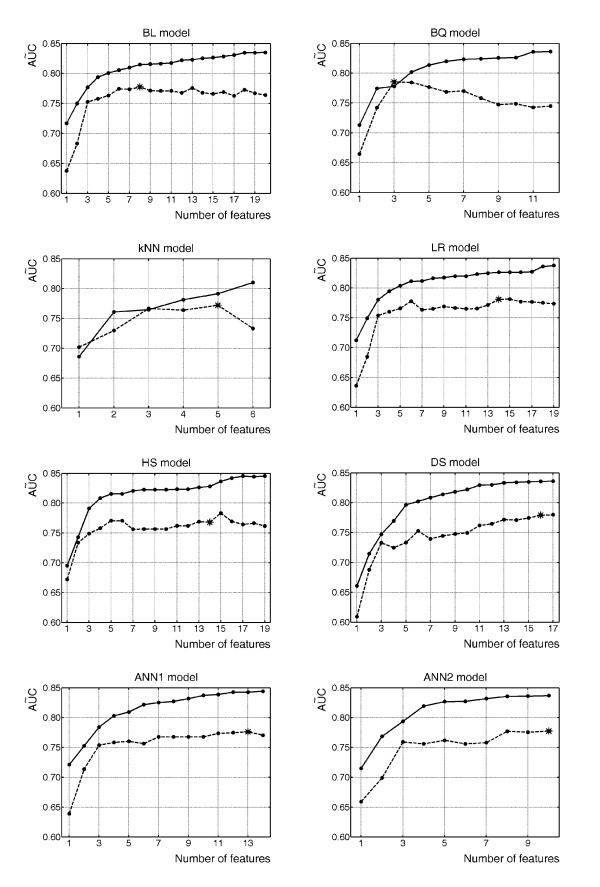
Median values of AUC (AŨC) obtained for the eight models by the bootstrap resampling method, in relation to the dimension of each best subset of features identified by the stepwise selection procedure. AŨC patterns in the training and test data are shown as continuous and dashed lines, respectively. The asterisk on the curve indicates the point of the optimal set of features for predicting morbidity. BL, Bayes linear; BQ, Bayes quadratic; *k*NN, *k*-nearest neighbour; LR, logistic regression; HS, Higgins score; DS, direct score; ANN1, one-layer artificial neural network; ANN2, two-layer artificial neural network.

Figure 1 shows the median values of AUC, obtained for each model by the bootstrap resampling method, in training and testing data (continuous and dashed lines, respectively) in relation to the dimension of each best subset of features identified by the stepwise procedure on training data. Since AŨC was taken as a global index of discrimination capacity, the difference between training and testing AŨC values may be considered to evaluate model generalization as a function of the number of features in the model: the greater the difference, the greater the model overfitting. The asterisk on the curve indicates the point corresponding to the optimal set of features for predicting morbidity, that is, the minimum number of selected features ensuring AUC values not statistically different from those giving the highest AŨC in the testing bootstrap data.

Table 8 lists the above-defined optimal set of predictor variables model by model and Table 9 shows the corresponding model performance. Discrimination capacity is quantified by AŨC calculated on bootstrap data. For testing data, 95% confidence intervals (CI) of AUC and CI%, that is, the percentage ratio of CI width to AŨC, are also given. Generalization was evaluated as the percentage difference in AŨC between training and testing data. Calibration was assessed on testing data by p of the Hosmer-Lemeshov goodness-of-fit test using Ĉ-statistics (HL-p), so that an HL-p much greater than 0.05 indicated very good model calibration, while HL-p < 0.05 revealed poor model calibration.

**Table 8:** Optimal feature vectors selected by different models from bootstrap test data

**No**.	**BL**	**BQ**	***k*NN**	**LR**	**HS**	**DS**	**ANN1**	**ANN2**
1	O_2_ER	O_2_ER	Post-CI	O_2_ER	O_2_ER	SvO_2_	O_2_ER	O_2_ER
2	VCO_2_	DO_2_	O_2_ER	VCO_2_	VCO_2_	Card-ID	Card-ID	VO_2_
3	Card-ID	Card-ID	Card-ID	Card-ID	Card-ID	DO_2_	VO_2_	Card-ID
4	PVD		PVD	PVD	PVD	O_2_ER	PVD	PVD
5	TBU		TBU	TBU	TBU	EM	TBU	Gly
6	EM			EM	EM	BSA	Pre-CI	Gender
7	SAP			SAP	SAP	AD	EM	MVR
8	SaO_2_			Pre-CI	Pre-CI	CHF	WBC	Cr
9				WBC	WBC	MVR	Age	AVO_2_
10				SaO_2_	SaO_2_	MR	SaO_2_	Arrhy
11				PvO_2_	PvO_2_	PVD	AD	
12				AD	AD	Diab	P/F	
13				PaO_2_	PaO_2_	VCO_2_	PVS	
14				Cr	Cr	CABG-C		
15						PAH		
16						IABP		

BL, Bayes linear model; BQ, Bayes quadratic model; *k*NN, *k*-nearest neighbour model; LR, logistic regression model; HS, Higgins score system; DS, direct score system; ANN1, one-layer artificial neural network; ANN2, two-layer artificial neural network. Predictor variable abbreviations are indicated in Tables 1-6.

**Table 9:** Number of selected features and corresponding model performance

**Model**	**No. of features**	**Testing discrimination AŨC (CI, CI%)**	**Training discrimination AŨC**	**Generalization ΔAŨC%**	**Calibration HL-p**
BL	8	0.778 (0.722–0.831, 14.0%)	0.815	4.5%	0.65*
BQ	3	0.785 (0.738–0.832, 12.0%)	0.780	-0.6%	0.19*
*k*NN	5	0.772 (0.717–0.822, 13.6%)	0.792	2.5%	0.01*
LR	14	0.781 (0.721–0.830, 14.0%)	0.827	5.6%	0.29
HS	14	0.768 (0.714–0.821, 13.9%)	0.828	7.2%	<0.001*
DS	16	0.779 (0.727–0.830, 13.2%)	0.836	6.8%	<0.001*
ANN1	13	0.776 (0.715–0.827, 14.4%)	0.843	7.9%	0.07*
ANN2	10	0.778 (0.726–0.825, 12.7%)	0.837	7.0%	0.01*

*after recalibration

BL, Bayes linear; BQ, Bayes quadratic; kNN, k-nearest neighbour; LR, logistic regression; HS, Higgins score; DS, direct score; ANN1, one-layer artificial neural network; ANN2, two-layer artificial neural network; AŨC, median value of area under receiver operating characteristic curve calculated from 1000 bootstrap samples; ΔAŨC%, difference between AŨC of training and test data; CI and CI%, confidence interval and percentage confidence interval; HL-p, p-value of the Hosmer-Lemeshow goodness-of-fit test.

Most models selected more than ten features to predict morbidity in the ICU after heart surgery (Table 8). The DS model used the largest set of features (sixteen predictor variables), while the number of features used in the HS model was set equal to that chosen by the corresponding LR model, as proposed by Higgins and colleagues [3]. The Bayesian and *k*NN models were much more parsimonious, using less than ten features. The Bayes quadratic model required the smallest set of predictor variables (only three).

Artificial neural networks gave the highest values of AŨC on training data, but their discrimination ability decreased sharply when estimated on testing data (Table 9). This result confirms that model overfitting may be a limitation of this approach.

The Bayes quadratic model, using only three predictor variables, provided the highest AŨC on test data (Table 9). Although the 95% confidence intervals of different models were largely superimposed, the Wilcoxon matched-pairs signed-ranks test on testing bootstrap data showed significant AUC differences between various models. This means that, when the results obtained with the bootstrap data were considered couple-by-couple, one model generally gave AUC values better than another. However, despite this statistical outcome, all models showed essentially not very dissimilar discrimination capacities, because the AŨC and CI were roughly equivalent from a practical point of view for the whole group of models. All models had acceptable discrimination capacities on test data, because their AŨC was always greater that 0.7 and less than 0.8 [11]. Furthermore, the width of the CI indicated appreciable sample variability in model discrimination performance.

All models showed satisfactory generalization when evaluated in our specialized ICU, because the percentage difference in AŨC between training and testing data was always less than 8% (Table 9). However the Bayes quadratic and *k*NN models had very good generalization performance, while artificial neural networks and integer score models gave the worst results.

The Hosmer-Lemeshov goodness-of-fit test indicated very poor calibration for both integer score models, even after recalibration (Table 9). However, this may also depend on limitations of the HL test in assessing goodness of fit for predictive models with discrete output probabilities. The *k*NN model and artificial neural networks (especially ANN2) also showed poor calibration, while the Bayesian and logistic regression models gave satisfactory results. Nevertheless, the Bayesian models had to be recalibrated, whereas the logistic regression model did not.

## Discussion

A pool of 78 variables was taken a priori as potential predictors of morbidity in the ICU after heart surgery, so that feature selection had to be made a posteriori, considering not only training but also testing data. Although some identical features were selected from all models, the number of predictor variables identified as optimal was rather different in the various models under study. As shown in Table 8, the Bayes quadratic model was the most parsimonious. Most other models (such as integer score systems) required many more predictors.

The DS model used the largest set of predictor variables. Table 7 shows that some features were entered in this model several times during the stepwise selection procedure: oxygen extraction (O_2_ER) was the most selected and obtainied the highest associated score. Despite a clear tendency to overfit training data (the differences between training and testing curves in Figure 1 increased remarkably with just a few features) AŨC significantly increased in test data with the number of selected features, reaching a value of 0.779 with sixteen predictors. Unfortunately this model showed very poor calibration performance, although this result may be partly due to the limitations of the HL test or the recalibration procedure for score models with discrete outputs. Furthermore, like other predictive score models, the DS system was difficult to update with new data. In fact, updating requires a complete periodic retraining. To do this, an automatic routine can be implemented on a computer, but this defeats the choice of this simple method which does not require a computer for everyday clinical application, the reason why such systems are very popular in medicine [3,6].

About the same number of features were selected for the LR model and ANN1. Most of the predictor variables selected by both on our ICU experimental data were the same (see Table 8). From a theoretical point of view, these two models are characterized by the same input-output nonlinear mathematical relationships, although their parameters are estimated by different approaches (for details see PartI of this study). This may justify the likeness of their discrimination results. However ANN1 performed better on training data and worse on test data, so that its generalization power was lower than that of the LR model, confirming the tendency of artificial neural networks to overfit training data. Much better results were also obtained by the LR model as regards calibration. Finally, difficulties can arise when designing and using artificial neural networks and continuous updating is practically impossible. These considerations suggests that the LR model is preferable to ANN1 for the example considered here.

The results obtained using the two-layer artificial neural network ANN2 were similar to those of ANN1, although ANN2 used a smaller feature set. Despite increased model complexity, ANN2 showed only slightly better discrimination on test data and generalization power, but worse calibration. So, when comparing ANN2 and LR performance, the same conclusion as between ANN1 and LR was reached.

As described in detail in PartI of this study, the Higgins score system was derived from the logistic regression model with the same features, by transforming continuous predictors to binary variables and LR coefficients to integer scores. Of course, the HS system suffers from the weaknesses of all integer score systems, as discussed when considering the DS model. Furthermore, the comparison of the results obtained by the LR and corresponding HS models showed that LR had better performance than the corresponding scoring system. In fact, its discrimination ability was higher on testing data and its generalization power was superior. The HS model showed very poor calibration, whereas the LR model was well calibrated even without any recalibration procedure. All this confirms that, when transforming the LR model into a simpler-to-use score system, it is necessary to carefully consider the cost of increased computational facility.

The Bayes linear model selected only eight features versus fourteen of the logistic regression model. The LR model used all the predictors used in the BL model and six additional ones. However, the number of model parameters estimated by the LR model was much less than that of BL: fifteen and fifty-two, respectively (see also Part I of this study). Despite these, the 95% confidence interval of AUC was the same for both models ([0.722–0.831] for BL versus [0.721–0.830] for LR) and the generalization power was similar. They both showed good calibration performances. This seems to confirm previous experimental findings indicating that in many practical situations the two approaches give generally similar results [6,18]. Their application in clinical practice is not difficult. To recognize morbidity a hand calculator is sufficient, because LR uses a simple exponential relationship and the Bayesian linear decision rule can be expressed as a linear function of the observation vector [7]. Major differences can be observed for updating. The BL model can be updated with new training data simply by updating the mean vector and pooled within-sample covariance matrix estimates using simple recursive relationships, whereas the LR model is not so simple to update. We therefore judged the BL model as better than LR in the present illustrative example.

The *k*NN model required only five features to predict morbidity in ICU patients after heart surgery. In general, this non parametric approach did not overfit training data, so that good generalization could also be obtained using different dimensions of the feature set (see Figure 1). In fact, generalization power only decreased appreciably with six predictor variables, that is, the maximum number of features selected by the stepwise procedure on training data. However its calibration was poor and AŨC computed on test data was the second last of all the models considered. Besides its computational cost and need for large data storage made this model unpromising, unless comparison of test cases with their *k *neighbours is considered important for comparative diagnosis.

The Bayes quadratic model had the highest discrimination capacity on test data, using the minimum number of features (oxygen extraction, oxygen delivery and need for cardiac inotropic drugs after the operation). AŨC calculated by means of the bootstrap resampling method was almost the same for training and testing data (percentage difference less than 1%). The quality of the results in the scenario considered may also be due to the small number of parameter estimates required by the model. In fact, with three predictor variables and two classes, the BQ model required the estimation of eighteen parameters (mean vectors and covariance matrices of the two classes). This model parameter number is about the same as that of the LR model (fifteen model parameters), but much less than that of BL (fifty-two model parameters). Like the BL model, the BQ model can be recursively updated whenever a new case has to be included in the training set. Finally, after recalibration, Hosmer-Lemeshov goodness-of-fit test using Ĉ-statistics indicated adequate model calibration. These considerations make the BQ model a convincing alternative to the BL and LR approaches for the present application.

It can be noted that two of the three predictors selected by the Bayes quadratic model (oxygen extraction and need for cardiac inotropic drugs after the operation) were chosen by all models. This means that these two variables were essential features for predicting morbidity in the scenario considered. Of course, the need for inotropic drugs after the operation is strongly correlated with poor cardiac function, while the key role played by oxygen extraction confirms the results of a previous study, in which increased oxygen extraction immediately after heart surgery has been indicated as an independent predictor of prolonged ICU stay [19]. The third predictor used by the BQ model was oxygen delivery and inadequate oxygen delivery has also been associated with prolonged ICU stay after heart surgery [20]. Increased levels of oxygen delivery and consumption have also been associated with improved outcome [21] and this fact has been tested in various clinical situations [22-24]. Knowledge of oxygen extraction and oxygen delivery is fundamental for assessing the relationship between oxygen consumption and oxygen delivery [25], though in many cases, mixed venous oxygen saturation or even central venous oxygen saturation alone may suffice [26]. Previous studies have shown that when oxygen saturation in the superior vena cava is used as a guide, early goal-directed therapy may provide significant benefits for outcome in ICU patients with severe sepsis and septic shock [27] and that this approach may reduce the length of hospital stay and the degree of organ dysfunction of heart surgery patients at discharge [28].

**Figure 2:**
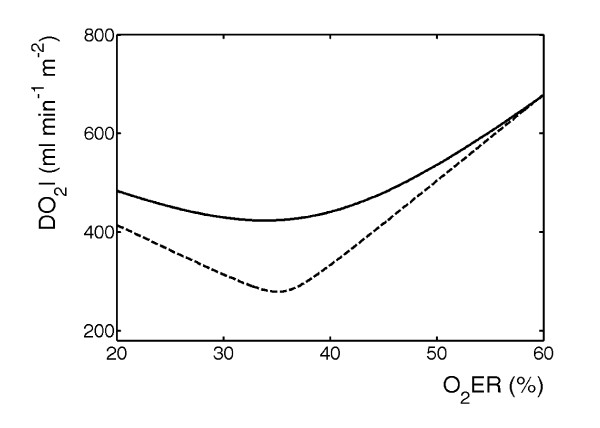
Decision boundaries separating morbid and normal course classes in the oxygen extraction/oxygen delivery plane for patients to whom cardiac inotropic drugs were (continuous line) and were not (broken line) administered. Patients at risk for morbidity are located below the decision boundary.

Statistical predictive models and artificial neural networks are black-box systems allowing cases to be allocated to different classes, but they do not lend themselves to interpretation of the underlying causes. However, when the number of the selected predictor variables is sufficiently small it may be interesting to seek an explanatory interpretation of the predictive model results a posteriori. In everyday life we are accustomed to considering phenomena in three dimensions. It is therefore difficult to expound the meaning of systems (such as predictive models) working in more than three dimensions. However, when the predictive model uses two or three features, a rational interpretation of its results may be attempted. The BQ model developed on our ICU data used only three features to predict morbidity outcomes, so that an interpretation of the result obtained was sought. First of all it is useful to recall that oxygen extraction is the ratio of oxygen consumption to oxygen delivery. A recent paper showed that the relationship of oxygen consumption to oxygen delivery is an important concept, even if its practical application is not simple and decisions regarding the need for strategies to increase and maintain oxygen delivery require the interpretation of many measurements [26]. The BQ predictive model seems to confirm these findings, because its decision boundary is given by a quadratic form of the three selected features in the three-dimensional space. In the clinical example used to locally develop the predictive model, this means that the cut-off value of oxygen delivery separating morbid and normal course classes does not remain constant or vary in a linear fashion as a function of oxygen extraction. Furthermore, this boundary changes in patients requiring cardiac inotropic drugs after the operation. Figure 2 clarifies this finding. Continuous and broken lines represent the decision boundaries in the oxygen extraction/oxygen delivery plane for patients to whom cardiac inotropic drugs are and are not administered, respectively. Patients at risk of morbidity are located below the decision boundary. The decision boundary moves up for patients who require drug administration after the operation, indicating that for these patients, the risk of morbidity will be high even at higher values of oxygen delivery.

As a conclusion, the Bayes quadratic model seemed to identify a minimum core of predictor variables generally recognized as essential for a pragmatic evaluation of the risk of morbidity after heart surgery. When this set of predictors was used on test data, it gave good discrimination, generalization and calibration, which were similar or better than those obtained with the Bayes linear or logistic regression models. Because of the small number of predictors to be monitored, clinicians may also more easily track and rationally interpret time courses of patient status, and consequently make prompt decisions about optimal therapeutic strategies. Of course, this does not mean that the Bayes quadratic approach is always the best model for predicting morbidity in ICU patients. However it provided a good compromise between system complexity and predictive performance in our example.

## Conclusion

The purpose of the present study was to analyse and compare different predictive models for estimating patient morbidity in the ICU after heart surgery. In this second part of the study we developed and tested eight popular predictive models with preoperative, intraoperative and postoperative data acquired in adult patients who underwent coronary artery bypass grafting. This part of the study supplements Part I in which different approaches for developing predictive morbidity models were reviewed in a unitary framework from a theoretical point of view.

The experimental results indicated that all models provided acceptable discrimination in test data and satisfactory generalization in our illustrative example. On the contrary poor calibration was obtained with scoring systems, the *k*-nearest neighbour model and artificial neural networks, while Bayes and logistic regression models gave satisfactory results. Most of models selected more than ten features to predict morbidity. Scoring systems and logistic regression model required the largest set of predictors, while Bayesian and *k*NN models were much more parsimonious, requiring less than ten features.

The Bayes quadratic model required the smallest set of predictor variables (only three: oxygen extraction, oxygen delivery and use of cardiac inotropic drugs after the operation) and provided very interesting results, which were similar or better than those obtained with the Bayes linear or logistic regression models. Unlike logistic regression models, an additional intrinsic strength of Bayesian models is that they can be updated in a straightforward manner, including new correctly classified cases into the training set, since this just involves the updating of mean vector and covariance matrix estimates by means of simple recursive relationships.

Because of the small number of predictors needed, the Bayes quadratic linear model also enabled an explanatory interpretation of the results obtained in our example. In particular, the BQ model seemed to confirm previous experimental findings proving that the relationship between oxygen consumption and oxygen delivery is a key issue for guiding therapy.

In conclusion, both theoretical and experimental findings indicate that the Bayes quadratic model offers a good compromise between complexity and predictive performances and can therefore be a convincing alternative to other much more extensively used predictive models (such as scoring systems or even Bayes linear and logistic regression models) in many clinical applications.

**Note: **This paper is accompanied by Part I, which gives a comprehensive review of several methods used to plan predictive models [29].

## Abbreviations

ANN = artificial neural network; AUC = area under the ROC curve; AŨC = median value of AUC, BL = Bayes linear; BQ = Bayes quadratic; CI = confidence interval; DS = direct score; HL = Hosmer-Lemeshow; HS = Higgins score; ICU = intensive care unit; *k*NN = *k*-nearest neighbour; LR = logistic regression; MSE = mean squared error; ROC = receiver operating characteristic.

## Competing interests

The author(s) declare that they have no competing interests.

## Authors' contributions

All authors participated in the study plan and coordination. GC and PB were concerned with medical informatics and biostatistical aspects of the study. EB was concerned with epidemiology and biostatistical aspects of the study. SS, BB and PG were involved in clinical aspects. SS collected clinical data. All authors read and approved the final manuscript.

## Pre-publication history

The pre-publication history for this paper can be accessed here:


